# Reports of Cases

**Published:** 1898-11

**Authors:** 


### REPORTS OF CASES.

### CÆSAREAN SECTION.[1]

By F. F. Hoffman, V.S..
BROOKSVILLE, PA.

The animals on which I have most frequently performed the operation are the sow and the bitch.

The most important point in this operation depends upon the manner in which we open the uterus. As a rule, these cases come to us after the patient has been in labor for two or three days and many efforts have been made by the owners and others to deliver the young. At such times we usually find our patient exhausted, the external organs lacerated and swollen, and part of a dead fœtus engaged in the vaginal canal, thus leaving but one chance, and that by operation.

Mode of procedure: I chloroform the patient, clip or shave the parts, scrub well with antiseptics; make my incision through the flank, pass the hand in, and draw the fœtus back. I then empty the bladder, after which I draw the uterus out and make the incision through the body, parallel with the bloodvessels, not across them, and thus avoid hemorrhage and laceration. The removal of the fœtus is now easy. Wash out the uterus and close with carbolized suture. Sponge out the abdominal cavity in the usual way and dress the wound antiseptically. The after-treatment of the case depends upon the condition of the patient. Five were operated upon during the past summer; one died under chloroform. Out of seventeen cases, consisting of sows, bitches, and cats, four have died.

---

The Commissioners of the District of Columbia will ask for three veterinarians next year: one for the police, fire, and engineer departments, and two for the health department, to act as inspectors of livestock and of dairy-farms. Surely no better evidence of an intelligent conservation of the health and comfort of the residents of the district could be exhibited.

[1] Abstract of report read at the semi-annual meeting of the Pennsylvania State Veterinary Medical Association.

